# Integration of Darolutamide in the Treatment Landscape for Metastatic Hormone-sensitive Prostate Cancer: A Systematic Review and Network Meta-analysis of Efficacy and Safety

**DOI:** 10.1016/j.euros.2025.11.011

**Published:** 2025-12-04

**Authors:** Felix Melchior, Magdalena Koett, Felix Keller, Nastasiia Artamonova, Giulia Giannini, Mona Kafka, Michael Ladurner, Hannes Neuwirt, Jasmin Bektic, Wolfgang Horninger, Isabel Heidegger

**Affiliations:** a Department of Urology, Medical University of Innsbruck, Innsbruck, Austria; b Department of Internal Medicine IV, Medical University of Innsbruck, Innsbruck, Austria

**Keywords:** Metastatic hormone-sensitive prostate cancer, Androgen receptor pathway inhibitors, Darolutamide, Doublet therapy, Triplet therapy, Treatment-emergent adverse events, Network meta-analysis, Systematic review

## Abstract

**Background and objective:**

Recent advances have led to the introduction of multiple combination treatments for metastatic hormone-sensitive prostate cancer (mHSPC), but their comparative efficacy and toxicity remain uncertain owing to the absence of head-to-head comparisons. We evaluated the efficacy and safety profile of darolutamide plus androgen deprivation therapy (ADT) in comparison to other treatments.

**Methods:**

A systematic search was conducted in the Cochrane Library up to September 30, 2024 in accordance with the Preferred Reporting Items for Systematic Reviews and Meta-Analyses guidelines. Hazard ratios (HRs) and confidence intervals (CIs) for progression-free survival (PFS) and overall survival (OS) were extracted. Odds ratios (ORs) for treatment-emergent adverse events (TEAEs) were calculated from the events reported.

**Key findings and limitations:**

Eleven trials involving 11 389 patients were included. Darolutamide triplet therapy was associated with the highest overall PFS (HR 0.24, 95% CI 0.20–0.29) and OS (HR 0.54, 95% CI 0.44–0.66). The highest PFS in low-volume disease was observed with enzalutamide (HR 0.29, 95% CI 0.21–0.38) and darolutamide (HR 0.30, 95% CI 0.15–0.60). All androgen receptor pathway inhibitors (ARPIs) had higher toxicity than ADT, except for darolutamide (OR 0.99, 95% CI 0.71–1.39). In comparison to darolutamide, enzalutamide (OR 2.03, 95% CI 1.08–3.80) and abiraterone (OR 3.18, 95% CI 1.74–5.80) were associated with higher risk of hypertension. Enzalutamide was associated with a higher risk of fatigue (OR 3.22, 95% CI 1.28–8.07). Limited direct comparisons between treatments may affect our conclusions regarding relative efficacy.

**Conclusions and clinical implications:**

Our findings support the role of darolutamide as an effective and well-tolerated ARPI for mHSPC, particularly in low-volume metachronous disease and comorbidity-limited cases. These results may assist clinicians in planning personalized treatment strategies that balance efficacy and safety.

**Patient summary:**

We compared different medical treatment options for metastatic hormone-sensitive prostate cancer, with a special focus on a drug called darolutamide. Darolutamide is well tolerated and is effective, particularly in patients with a low volume of metastasis and patients with other health conditions that may limit their treatment options.

## Take Home Message

Triplet therapy combinations offer superior survival outcomes in metastatic hormone-sensitive prostate cancer. Among doublet combinations, darolutamide-based therapy provides competitive efficacy with a favorable safety profile. Treatment decisions should therefore be personalized beyond disease volume alone in shared decision-making between patients and clinicians.

## 1 Introduction

Prostate cancer ranks among the most prevalent malignancies worldwide, representing the second most common cause of cancer-related mortality in the male population [1]. Up to 20% of patients present with de novo metastatic disease, while others experience metachronous metastasis after primary local treatment [2], [3]. Androgen deprivation therapy (ADT) has historically been the foundational systemic treatment for metastatic hormone-sensitive prostate cancer (mHSPC), serving as the standard of care (SOC) for decades [4]. However, in recent years, the treatment paradigm for mHSPC has evolved significantly with the introduction of innovative therapies, including chemotherapy and androgen receptor pathway inhibitors (ARPIs) [5], [6], [7], [8], [9], [10], [11]. These advances have expanded the therapeutic arsenal for mHSPC but have also increased the complexity of clinical decision-making [12]. This complexity arises from the need to balance multiple considerations, including the relative efficacy of treatments, associated side effects, cost implications, patient-specific factors such as performance status, and drug accessibility across different health care settings. Consequently, selection of the optimal treatment strategy necessitates a nuanced, individualized approach that incorporates both clinical evidence and patient preferences.

Of importance, first results from the ARANOTE trial were presented at the European Society for Medical Oncology (ESMO) meeting in October 2024, and demonstrate that darolutamide + ADT increased radiographic progression-free survival (rPFS) in comparison to ADT + placebo [13]. While meta-analyses published up to October 2024 compared the effectiveness of various mHSPC treatments [14], [15], comprehensive data from ARANOTE were not included. Thus, we conducted a systematic review and network meta-analysis (NMA) of phase 3 mHSPC trials, with a focus on ARANOTE to contextualize the comparative efficacy and safety of darolutamide + ADT within the spectrum of treatment regimens available. By synthesizing the latest evidence, our aim was to provide further insights for clinicians to support shared decision-making and optimize the integration of darolutamide + ADT into individualized treatment strategies. While our study aligns with recent data regarding efficacy, our focused analysis of toxicity provides novel insights, and addresses a critical gap in the literature and advancing comprehension in this domain.

## 2 Methods

Our NMA followed the Preferred Reporting Items for Systematic Reviews and Meta-Analyses (PRISMA) guideline [16], [17]. The study protocol was registered a priori in the International Prospective Register of Systematic Reviews database (PROSPERO: CRD42024604751).

### 2.1 Data sources and search strategy

The Cochrane Library, one of the most extensive databases on this topic, was searched to identify reports on systemic therapy for mHSPC. The search focused on phase 3 clinical trials published between January 2014 and September 2024. Search parameters encompassed primary terms such as prostate cancer, hormone- or castration-sensitive, metastatic or advanced, and randomized. The exact keywords used in our search strategy are presented in Supplementary Fig. 1.

### 2.2 Inclusion criteria

The inclusion criteria comprised phase 3 clinical trials assessing treatment with ADT in combination with ARPIs, chemotherapy, or placebo in patients with mHSPC. Studies published in English between January 2014 and September 2024 were included. Trials involving any treatments other than those listed in the inclusion criteria were excluded. Reviews, meeting abstracts, letters, editorials, observational studies, and case reports were also excluded. Reference lists in all the papers included were scanned for additional studies of interest.

### 2.3 Data extraction and assessment of study quality

Two authors (F.M. and M.K.) independently extracted data and resolved discrepancies via consensus. The study name and title, first author’s name, publication year, preliminary therapies, number of patients, tumor status, treatment dosage, reference substances, median age, median PSA, median Gleason score at initial diagnosis, disease volume, tumor risk group, Eastern Cooperative Oncology Group (ECOG) performance status [18], outcome, median follow-up duration, and treatment-emergent adverse events (TEAEs) were extracted. Hazard ratios (HRs) with 95% confidence intervals (CIs) and *p* values associated with the outcomes of interest were retrieved. Two senior authors checked the extracted data for completeness and accuracy. The risk of bias for each study was assessed independently by two authors via the Cochrane Risk of Bias 2 (RoB 2) tool (Supplementary Fig. 2) [19]. The certainty of evidence for each outcome was assessed using the Grading of Recommendations Assessment, Development and Evaluation (GRADE) framework (Supplementary Table 1) [20], [21].

### 2.4 Main outcomes

The joint primary endpoints were PFS and overall survival (OS). PFS was defined as the time from randomization to either clinical progression (cPFS) or radiographic progression (rPFS) or death. OS was defined the time from randomization to death from any cause. Subgroup data comprised age groups (<65–70 vs >70–75 yr), ECOG performance status, disease volume defined according to the CHAARTED criteria [5], and tumor status at diagnosis. Secondary endpoints included safety and toxicity, in terms of the incidence and severity of overall TEAEs. In particular, the occurrence of hypertension, fatigue and, bone fractures was assessed. Common Terminology Criteria for Adverse Events (CTCAE) v5.0 was used to grade the severity of TEAEs [22].

### 2.5 Statistical analysis

We performed frequentist NMAs of treatment efficacy and adverse events using random-effects models. This choice reflects the assumption that true treatment effects might differ across studies because of variations in patient populations, interventions, and study methods, even if such heterogeneity was not statistically significant. However, we recognize that random-effects models do not resolve or explain heterogeneity. Accordingly, we explored possible sources of heterogeneity by examining clinical and methodological differences among the studies included, rather than relying solely on statistical models to address variability. For treatment efficacy endpoints, HRs were used to estimate the relative risk. In cases in which standard errors were not reported, CIs were extracted and transformed into the standard error of the log HR to reflect uncertainty in the inverse variance–weighted NMA. For TEAEs, odds ratios (ORs) and their respective standard errors were calculated from the absolute event frequencies reported. The network included studies comparing two or more interventions directly or indirectly through a common comparator. A network diagram was constructed to visualize the relationships among interventions, with nodes of the graph representing interventions and edges indicating the direct comparisons available. ADT was the most central common comparator and served as the reference treatment. Statistical heterogeneity was assessed using the τ^2^ statistic and *p* values for Cochran’s Q. Inconsistency and sensitivity were further addressed via subgroup analyses using commonly reported strata. Results are presented as forest plots showing the relative risk with 95% CI in comparison to the reference treatment, and measures of heterogeneity. We allowed for a type 1 error of 5%. All analyses were performed using R v4.4.2 [23].

## 3 Results

### 3.1 Study selection and baseline characteristics

Following abstract screening and full-text review, 11 trials met our predetermined inclusion criteria (Table 1). Including both interim and final analyses as well as subgroup analyses, 18 full texts were analyzed. The network of evidence comprised seven different treatments: docetaxel, abiraterone, enzalutamide, apalutamide, darolutamide, darolutamide + docetaxel, and abiraterone + docetaxel, each added to ADT. Abiraterone was administered in combination with prednisolone, hereafter referred to as abiraterone + ADT. A total of 11 389 patients were included across all the studies (5622 in the experimental group vs 5767 in the control arm). The network diagram illustrating the geometry of the treatment comparisons for the overall population is presented in Supplementary Fig. 3. Forest plots of the direct comparisons for PFS, OS, and TEAEs are shown in Supplementary Figs. 6–8.

**Table 1:** Baseline characteristics of the trials included in the network meta-analysis

	GETUG-AFU15	STAMPEDE(arm C)	STAMPEDE (arm G)	CHAARTED	LATITUDE	ARCHES	ENZAMET	TITAN	PEACE-1	ARASENS	ARANOTE
Year	2015	2016	2017	2018	2019	2019	2019	2019	2022	2022	2024
Combination agent	Doc	Doc	Abi	Doc	Abi	Enza	Enza	Apa	Abi + Doc	Daro + Doc	Daro
ECOG PS	≤2	≤2	≤2	≤2	≤2	≤1	≤2	≤1	≤2	≤1	≤2
Disease stage	mHSPC	DN mHSPC	DN mHSPC or HR-laPC	mHSPC	DN high-risk mHSPC	mHSPC	mHSPC	mHSPC	DN mHSPC	mHSPC	mHSPC
Previous treatment	ADT	ADT	ADT	ADT	ADT or ORC + FGA or palliative S/RT	ADT ± Doc	ADT ± Doc	Doc or ADT or local/palliative S/RT	ADT	ADT ± FGA	ADT ± FGA
Prior docetaxel C/E (%)	No	No	No	No	No	18/18	15/17	10/11	No	No	No
Study population (*n*)	385	1086	1917	790	1199	1150	1125	1052	710	1306	669
Primary endpoint	OS	OS	OS	OS	OS + PFS	rPFS	OS	OS + rPFS	OS + rPFS	OS	rPFS
mFU (mo)	84	78	40	53.7	30.4 (51.8)^a^	14.4 (44.6)^a^	34	22.7 (44.0)^a^	36/45.6^b^	42.4/43.7^c^	25.0/25.3^c^
**Control arm (*n*)**	**193**	**724**	**957**	**393**	**602**	**576**	**562**	**527**	**355**	**654**	**223**
Treatment	ADT	ADT	ADT	ADT	Pbo + ADT	Pbo + ADT	FGA + ADT	Pbo + ADT	Doc + ADT	Doc + ADT + Pbo	Pbo + ADT
Median age, yr (range)	64 (58–70)^d^	65 (60–71)^d^	67 (62–72)^d^	63 (39–91)	67 (33–92)	70 (42–92)	69 (64–75)^d^	68 (43–90)	66 (59–70)	67 (42–86)	70 (45–91)
High/low DV (%)	47/43	44/56	57/43	64/36	78 /22	65/35	53/47	64/36	65/35	78/22	70/30
DN disease (%)	76	100	96	73	100	72	58	85	100	87	75
mPSA, ng/ml (range)	25.8 (5.0–126.9)^d^	103 (33–354)^d^	56 (19–165)^d^	52 (0–8056)	NR	5 (0–19 000)	NR	4 (0–2229)	12 (3–60)	30 (0–9219)	21 (0–8533)
GS ≥8 at iDx (%)	24	66	75	62	97	65	57	68	80	79	66
**Experimental arm (*n*)**	**192**	**362**	**960**	**397**	**597**	**574**	**563**	**525**	**355**	**651**	**446**
Treatment	Doc 75 mg/m^2^ + ADT	Doc 75 mg/m^2^ + ADT	Abi 1000 mg + ADT + Pdn 5 mg	Doc 75 mg/m^2^ + ADT	Abi 1000 mg + ADT + Pdn 5 mg	Enza 160 mg + ADT	Enza 160 mg + ADT	Apa 240 mg + ADT	Abi 1000 mg + ADT + Doc	Daro 1200 mg + ADT + Doc	Daro 1200 mg + ADT
Median age, yr (range)	63 (57–68.2)^d^	65 (60–70)^d^	67 (63–72)^d^	64 (36–88)	68 (38–89)	70 (46–92)	69 (63–75)^d^	69 (45–94)	66 (60–70)	67 (41–89)	70 (43–93)
High/low DV (%)	48/42	41/59	46/54	66/34	82/18	62/38	52/48	62/38	63/37	76/24	71/29
*De novo* disease (%)	67	100	94	73	100	73	58	82	100	86	71
mPSA, ng/ml (range)	26.7 (5.0–106.2)^d^	97 (41–340)^d^	51 (19–158)^d^	51 (0–8540)	NR	5 (0–4824)	NR	6 (0–2682)	14 (2–59)	24 (0–11 947)	21 (0–15 915)
GS ≥8 at iDx (%)	32	70	74	61	98	67	60	67	77	78	70

Abi = abiraterone; ADT = androgen deprivation therapy; Apa = apalutamide; C/E = control/experimental arm; Daro = darolutamide; Doc = docetaxel; DN = de novo; DV = disease volume; ECOG PS = Eastern Cooperative Oncology Group performance status; Enza = enzalutamide; FGA = first-generation antiandrogen; GS = Gleason score; iDx = initial diagnosis; IQR = interquartile range; HR-laPC = high-risk locally advanced prostate cancer; mFU = median follow-up; mHSPC = metastatic hormone-sensitive prostate cancer; NR = not reported; ORC = orchiectomy; OS = overall survival; Pbo = placebo; Pdn = prednisolone; (r)PFS = (radiographic) progression-free survival; mPSA = median prostate-specific antigen; S/RT = surgery/radiation therapy.

a mFU (mFU for OS in final analysis).

b mFU for rPFS/OS.

c mFU for control arm/experimental arm.

d Interquartile range.

The studies were published between 2015 and 2024, with sample sizes ranging from 385 to 1917 patients. Median follow-up across the studies ranged from 25 to 84 months. Baseline characteristics of the patient populations were similar across the studies and are summarized in Table 1. Risk-of-bias assessment revealed moderate overall quality in seven trials, with downgrading primarily because of lack of blinding (Supplementary Fig. 2).

### 3.2 Network meta-analysis

#### 3.2.1 Progression-free survival

**Fig. 1:**
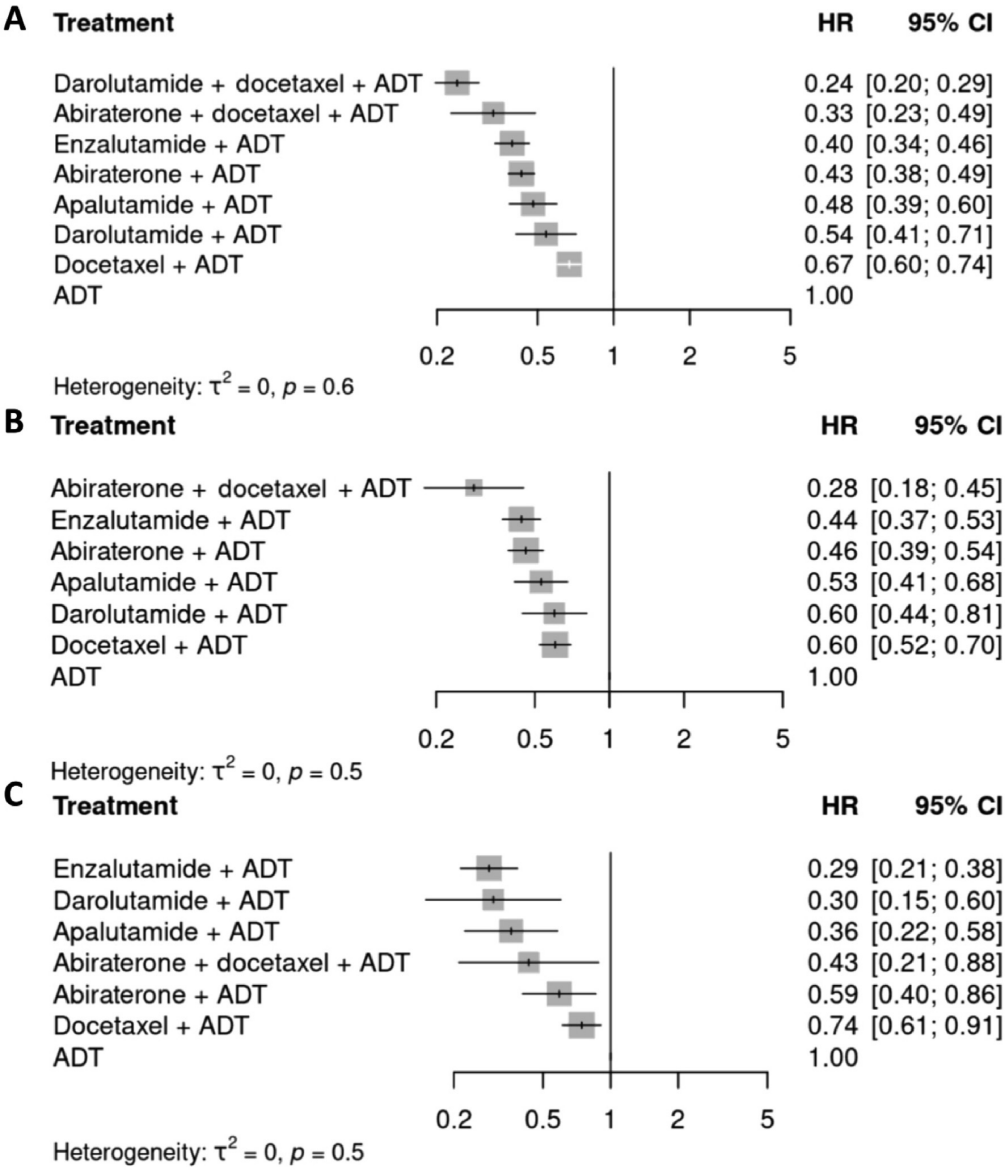
Forest plots of the hazard ratio (HR) for progression-free survival with various treatment combinations in comparison to androgen deprivation therapy (ADT) according to random-effects models for (A) the overall population and the groups with (B) high-volume disease or (C) low-volume disease. No data for subgroup analysis could be retrieved for darolutamide + docetaxel + ADT. CI = confidence interval.

Owing to missing data for PFS in the ARASENS trial, time to castration-resistant prostate cancer (ttCRPC) was used as a surrogate for PFS. Thus, no subgroup analysis data for PFS could be retrieved for this trial. A sensitivity analysis was performed to assess the robustness of this approach (Supplementary Fig. 4C). In comparison to SOC, all the treatments investigated resulted in significantly better PFS. Darolutamide combined with docetaxel and ADT was associated with the highest PFS (HR 0.24, 95% CI 0.20–0.29), followed by abiraterone combined with docetaxel and ADT (HR 0.33, 95% CI 0.23–0.49), and various ARPI doublet treatments thereafter (Fig. 1A).

Subgroup analysis revealed that all interventions significantly improved PFS in comparison to SOC independently of tumor burden (Fig. 1B, C). Of note, abiraterone combined with docetaxel and ADT was associated with the greatest PFS benefit in high-volume disease (HR 0.28, 95% CI 0.18–0.45). Enzalutamide + ADT (HR 0.29, 95% CI 0.21–0.38) followed by darolutamide + ADT (HR 0.30, 95% CI 0.15–0.60) were associated with the greatest PFS benefits in low-volume disease (Fig. 1C). Results for PFS subgroup analysis stratified by age are shown in Supplementary Fig. 4.

#### 3.2.2 Overall survival

**Fig. 2:**
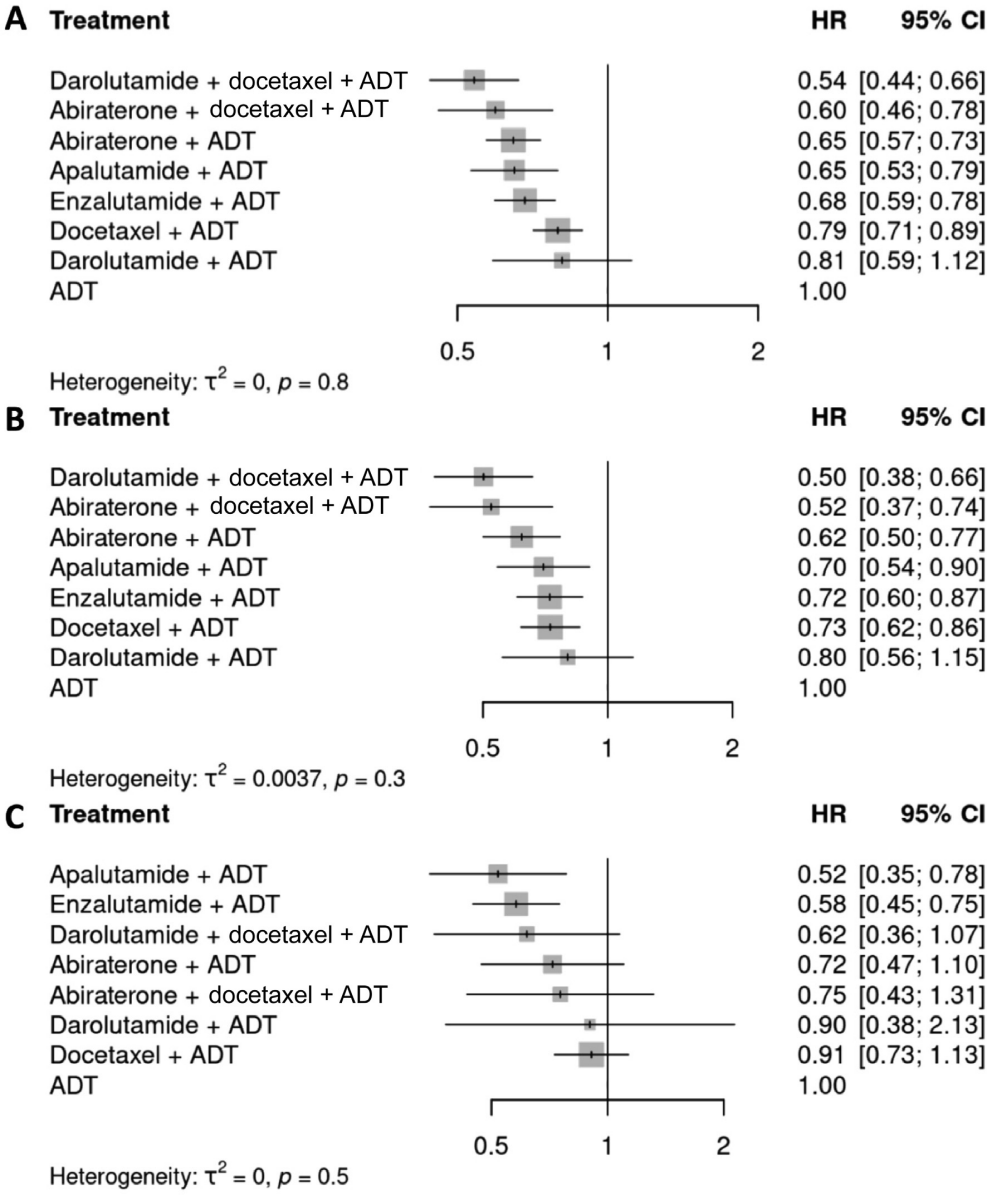
Forest plots of the hazard ratio (HR) for overall survival with various treatment combinations in comparison to androgen deprivation therapy (ADT) according to random-effects models for (A) the overall population and the groups with (B) high-volume disease or (C) low-volume disease. CI = confidence interval.

In comparison to SOC, all interventions except for darolutamide + ADT were associated with significant improvements in OS (Fig. 2A). However, the lack of apparent benefit for this combination may be attributable to the relatively short follow-up of 25 mo for ARANOTE, with immature OS data. Darolutamide combined with docetaxel and ADT had the lowest HR in both the overall population (HR 0.54, 95% CI 0.44–0.66) and the high-volume subgroup (HR 0.50, 95% CI 0.38–0.66), followed by abiraterone combined with docetaxel and ADT, and then various ARPI doublet treatments (Fig. 2A,B). Apalutamide + ADT was associated with the highest OS in low-volume disease (HR 0.52, 95% CI 0.35–0.78; Fig. 2C). Moreover, darolutamide combined with docetaxel and ADT was associated with an increase in OS independently of age, ECOG performance status, or the presence of metastases at initial presentation (Supplementary Fig. 5A–E).

#### 3.2.3 TEAEs and safety

**Fig. 3:**
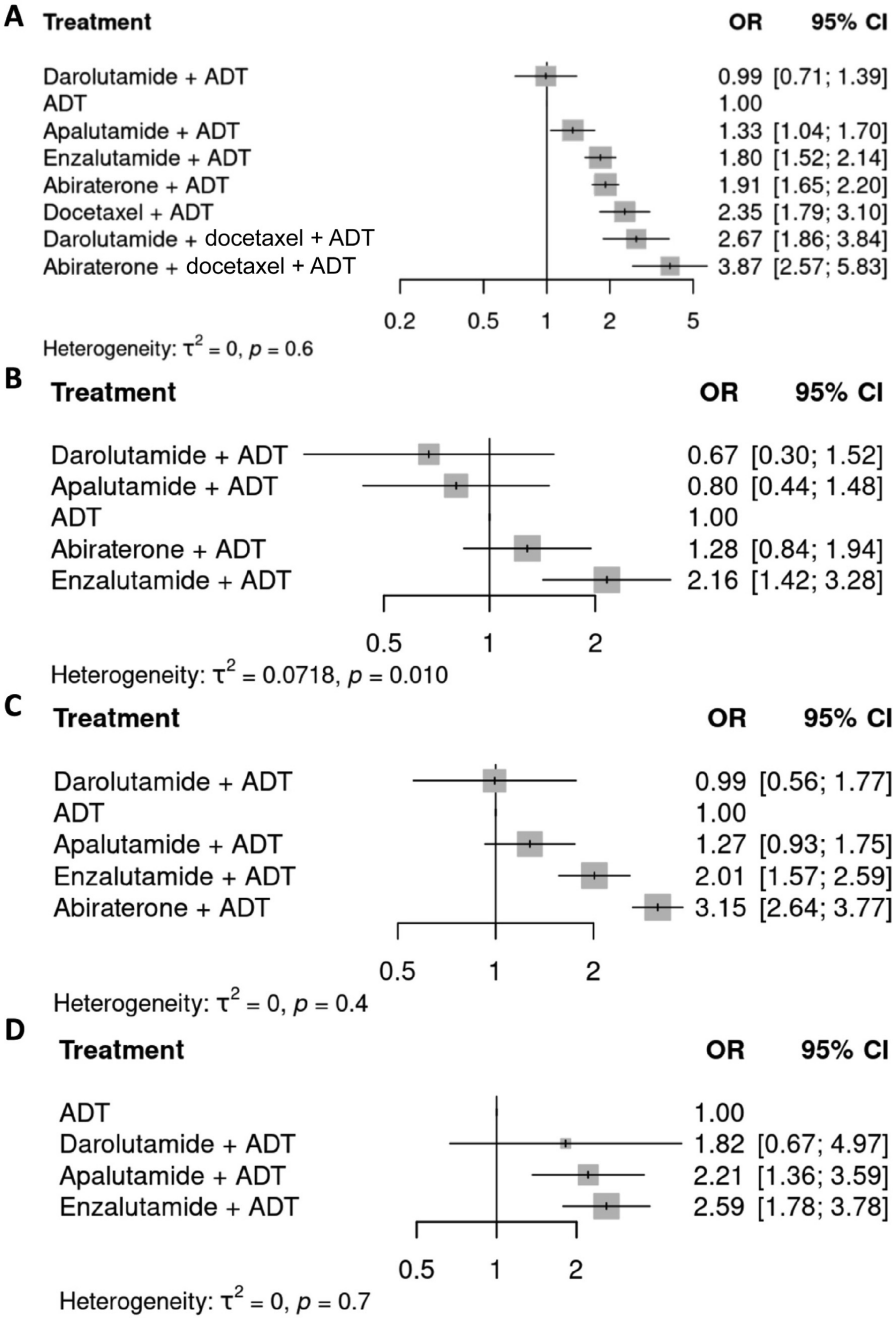
Forest plots of the odds ratio (OR) for treatment-emergent adverse events (TEAEs) with various treatment combinations in comparison to androgen deprivation therapy (ADT) according to random-effects models for (A) grade ≥3 TEAEs, (B) fatigue, (C) hypertension, and (D) bone fractures in the overall population. CI = confidence interval.

**Fig. 4:**
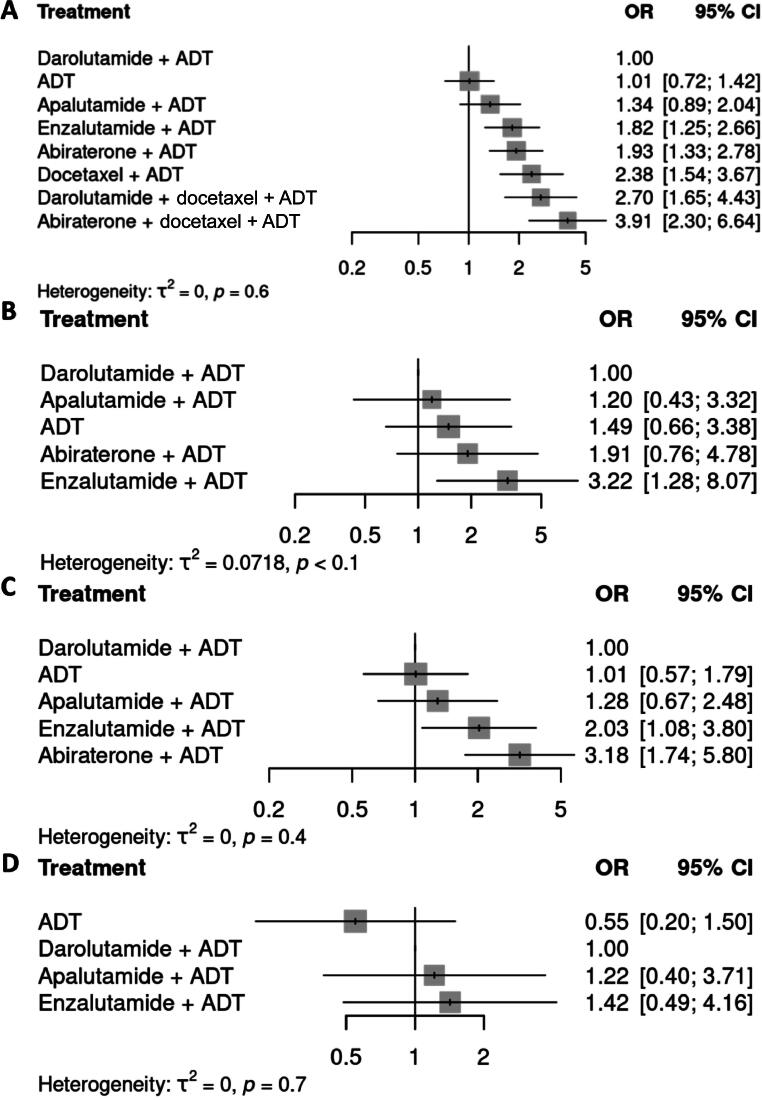
Forest plots of the odds ratio (OR) for treatment-emergent adverse events (TEAEs) with various treatment combinations in comparison to androgen deprivation therapy (ADT) + darolutamide according to random-effects models for (A) grade ≥3 TEAEs, (B) fatigue, (C) hypertension, and (D) bone fractures in the overall population. CI = confidence interval.

In terms of grade ≥3 TEAEs, all the ARPIs assessed were associated with a significantly higher risk of toxicity in comparison to ADT alone, except for darolutamide + ADT (Fig. 3A; OR 0.99, 95% CI 0.71–1.39). In comparison to darolutamide + ADT, all other ARPIs were associated with a significantly higher risk of toxicity, except for apalutamide + ADT (Fig. 4A; OR 1.34, 95% CI 0.89–2.04).

Enzalutamide + ADT was associated with a significantly higher risk of fatigue in comparison to both ADT alone (Fig. 3B; OR 2.16, 95% CI 1.42–3.28) and darolutamide + ADT (Fig. 4B; OR 3.22, 95% CI 1.28–8.07). While the risk of fatigue was lower with darolutamide + ADT (OR 0.67, 95% CI 0.30–1.52) and apalutamide + ADT (OR 0.80, 0.44–1.48) in comparison to ADT, the differences were not statistically significant. The risk of hypertension was significantly higher with enzalutamide + ADT (OR 2.01, 95% CI 1.57–2.59) and abiraterone + ADT (OR 3.15 95% CI 2.64–3.77) in comparison to ADT alone (Fig. 3C). The risk of hypertension was also significantly higher with enzalutamide + ADT (OR 2.03, 95% CI 1.08–3.80) and abiraterone + ADT (OR 3.18, 95% CI 1.74–5.80) in comparison to darolutamide + ADT (Fig. 4C). By contrast, darolutamide + ADT was not associated with significantly higher risk of hypertension in comparison to ADT alone (OR 0.99, 95% CI 0.56–1.77; Fig. 3C). Assessment of skeletal events revealed that the risk of bone fractures was significantly higher with apalutamide + ADT (OR 2.21, 95% CI 1.36–3.59) and enzalutamide + ADT (OR 2.59, 95% CI 1.78–3.78) in comparison to ADT alone (Fig. 3D). Darolutamide + ADT was not associated with significantly higher risk of bone fractures (Fig. 3, Fig. 4). No data on bone fractures could be retrieved for abiraterone + ADT.

## 4 Discussion

The past decade has seen significant transformation of treatment strategies for mHSPC, driven by the landmark CHAARTED and STAMPEDE trials [5], [24]. These pivotal studies demonstrated a substantial OS benefit with the combination of ADT + docetaxel, which challenged the historical reliance on ADT monotherapy as SOC. Since then, the therapeutic landscape has rapidly evolved with the advent of multiple dual-therapy regimens. The LATITUDE [25] and STAMPEDE (arm G) [26] trials established the efficacy of ADT combined with abiraterone, while ENZAMET [7] demonstrated the clinical benefits of ADT + enzalutamide. Similarly, TITAN [9] underscored the role of ADT + apalutamide, and, more recently, the ARANOTE trial in 2024 introduced robust evidence supporting ADT + darolutamide as an effective regimen [13]. Of importance, a paradigm shift emerged in 2022 on the basis of findings from the PEACE-1 and ARASENS trials [10], [11], which revealed superior efficacy of triplet therapies comprising ADT + docetaxel + an ARPI (abiraterone in PEACE-1; darolutamide in ARASENS) over the previously established dual regimen of ADT + docetaxel, primarily for patients with synchronous high-volume disease [27], [28].

Despite these advances, the lack of direct head-to-head comparisons of the various treatment regimens remains a critical limitation, especially when aiming for personalized treatment strategies. In this NMA, we synthesized evidence from 11 RCTs involving 11 389 patients to evaluate systemic therapies for mHSPC. To the best of our knowledge, this is the first NMA to incorporate the latest safety and efficacy data, including TEAE profiles, from both triplet and doublet therapies and, most notably, recent findings from the ARANOTE trial.

We also included long-term outcomes from pivotal studies such as LATITUDE, ARCHES, and TITAN [25], [29], [30], which provides a longitudinal perspective on treatment efficacy and safety. The aim of our approach was to bridge the gap left by the absence of direct comparisons and to provide a nuanced understanding of the evolving therapeutic landscape in mHSPC, insights that are crucial for evidence-based, personalized clinical decision-making.

### 4.1 Key findings

Regarding efficacy, addition of an ARPI or ARPI-chemotherapy combinations to ADT significantly improved PFS in comparison to ADT alone. Among triplet therapies, darolutamide-based triplet combinations resulted in the highest PFS and OS in the overall population, as well as the highest OS in high-volume disease. Regarding PFS, abiraterone triplet therapy resulted in the greatest benefit in high-volume disease. These findings align with a prior NMA by Mandel et al [31] that emphasized the PFS and OS advantages of triplet regimens in high-volume disease. Our study expands the evidence by including recent data from ARANOTE that underscore the efficacy of darolutamide in doublet combinations. While darolutamide + ADT significantly improved PFS, it did not lead to statistically significant OS benefits within the relatively short median follow-up in the trial. These results align with recently published data [32], [33]. It is worth mentioning that ARANOTE was not designed to demonstrate OS, as this had already been established in the ARASENS trial; nonetheless, the interim data already show a trend towards improving OS. This highlights the need for extended follow-up to fully assess the impact on long-term survival.

Our analysis underscores the favorable safety profile of darolutamide-based therapies. Importantly, darolutamide doublet therapy was the only treatment that did not significantly increase the risk of grade ≥3 TEAEs in comparison to ADT monotherapy. Darolutamide also resulted in lower incidence of side effects commonly associated with ARPIs, including fatigue, hypertension, and bone fractures, although these differences were not always statistically significant.

These findings are consistent with previous research, such as the ODENZA trial, which revealed lower fatigue levels and better episodic memory with darolutamide in comparison to enzalutamide [34]. In the ARAMIS trial, darolutamide was associated with only minimal increases in TEAEs, even for prolonged treatment [35], [36]. The molecular characteristics of darolutamide, including limited central nervous system penetration and a lower level of CYP450-mediated drug-drug interactions, presumably underpin its greater tolerability [37]. These pharmacological attributes make darolutamide an appealing treatment choice, particularly for patients at higher risk of treatment-related toxicities and those with significant comorbidities.

### 4.2 Limitations

While we believe that the key trials are well represented in our NMA, our systematic search was limited to the Cochrane Library, which may have limited the comprehensiveness of the evidence base. Furthermore, NMAs rely on indirect comparisons, so they are inherently observational and susceptible to biases not present in direct trials. Consequently, some degree of clinical and methodological variability among the studies included remains. For example, heterogeneity in patient populations and study design may influence outcomes. Particularly noteworthy are differences in the prevalence of de novo disease, which ranged from 67% to 100%, and recruitment from diverse geographic regions. Moreover, varying PFS definitions across studies—such as radiological progression, clinical progression, and ttCRPC—and the prior administration of docetaxel in ARCHES and TITAN must be acknowledged. NMA findings depend on the reporting quality of the trials included. RoB 2 assessment identified potential bias in several trials, which particularly affected the TEAE analysis. Open-label designs, as in PEACE-1 and ENZAMET, may have introduced performance and detection bias in TEAE reporting. ENZAMET also allowed concomitant docetaxel use in approximately 65% of enzalutamide patients, which potentially confounds TEAEs commonly associated with chemotherapy. Therefore, the safety and toxicity results should be interpreted accordingly. While our analysis incorporated long-term results from established trials, data on extended outcomes for newer treatments remain limited. Specifically, OS data from ARANOTE are not yet mature. Overall, these limitations underscore the importance of future research prioritizing direct head-to-head comparisons of the most promising treatments to address these gaps.

## 5 Conclusions

Our NMA provides a comprehensive synthesis of efficacy, safety, and toxicity data for mHSPC treatment regimens, with integration of the latest evidence from the ARANOTE trial presented at ESMO 2024.

These findings support individualized treatment selection for mHSPC, for which triplet therapy offers the greatest survival benefit in fit patients, particularly those with de novo high-volume disease. However, darolutamide-based doublet therapy appears to be particularly suitable for patients with metachronous low-volume disease, performance status limitations, pre-existing neurological conditions, concomitant anticoagulant therapy, higher risk of treatment-related toxicity, or contraindications to more intensive regimens, for whom the safety profile and minimal drug interactions of darolutamide could provide significant clinical advantages. While these findings do not support a universal treatment recommendation, they may assist clinicians in weighing comparative benefits and risks when tailoring treatment decisions. As individualized care remains the cornerstone of good oncologic practice, treatment selection must continue to be based on patient-specific factors, including prior treatments, disease burden, comorbidities, and personal preferences.

From a research perspective, our results need to be validated in prospective head-to-head trials that include long-term outcomes, quality-of-life measures, and cost-effectiveness analyses to better inform clinical decision-making.

  ***Author contributions:*** Isabel Heidegger had full access to all the data in the study and takes responsibility for the integrity of the data and the accuracy of the data analysis.

  *Study concept and design*: Heidegger, Melchior, Koett, Keller.

*Acquisition of data*: Heidegger, Melchior, Koett, Keller.

*Analysis and interpretation of data*: Heidegger, Melchior, Koett, Keller.

*Drafting of the manuscript*: All authors.

*Critical revision of the manuscript for important intellectual content*: Heidegger, Melchior, Koett, Keller.

*Statistical analysis*: Keller.

*Obtaining funding*: None.

*Administrative, technical, or material support*: Heidegger.

*Supervision*: Heidegger.

*Other*: None.

  ***Financial disclosures:*** Isabel Heidegger certifies that all conflicts of interest, including specific financial interests and relationships and affiliations relevant to the subject matter or materials discussed in the manuscript (eg, employment/affiliation, grants or funding, consultancies, honoraria, stock ownership or options, expert testimony, royalties, or patents filed, received, or pending), are the following: Isabel Heidegger, Jasmin Bektic, Mona Kafka, Giulia Giannini, Nastasiia Artamonova, and Michael Ladurner report speaker and consulting fees from Bayer, Astellas, and Johnson & Johnson. The remaining authors have nothing to disclose.

  ***Funding/Support and role of the sponsor:*** None.
