# Supplementary material for: Integration of Darolutamide in the Treatment Landscape for Metastatic Hormone-sensitive Prostate Cancer: A Systematic Review and Network Meta-analysis of Efficacy and Safety
Source: Eur Urol Open Sci. 2025 Dec 4;83:72–82. doi: 10.1016/j.euros.2025.11.011 (PMC12720369; doi:10.1016/j.euros.2025.11.011)
Supplement: Supplementary Data 1 [file mmc1.docx]

**Supplementary Appendix**

Data Sources and Search Strategy


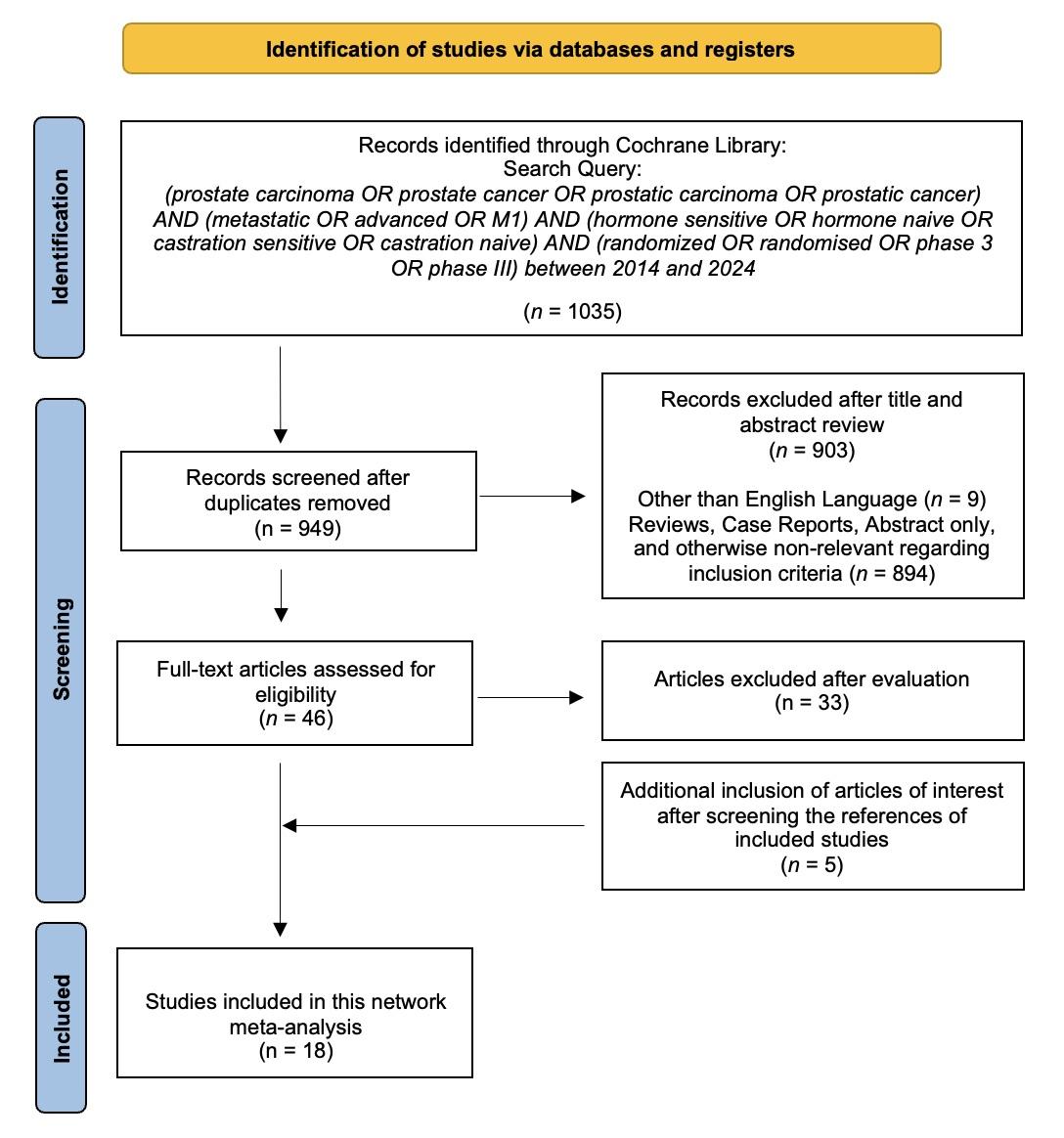


**Figure S1**: The PRISMA flow chart, detailing the study selection process.

Risk of Bias Assessment

This tool evaluates five domains: bias arising from the randomization process, deviations from intended interventions, missing outcome data, measurement of the outcome, and selection of the reported result. Two authors independently conducted the assessment, resolving discrepancies through discussion or consultation with a third author. Each domain was judged as having low risk, some concerns, or high risk of bias, with overall risk determined based on these judgments (Figure S2).

**
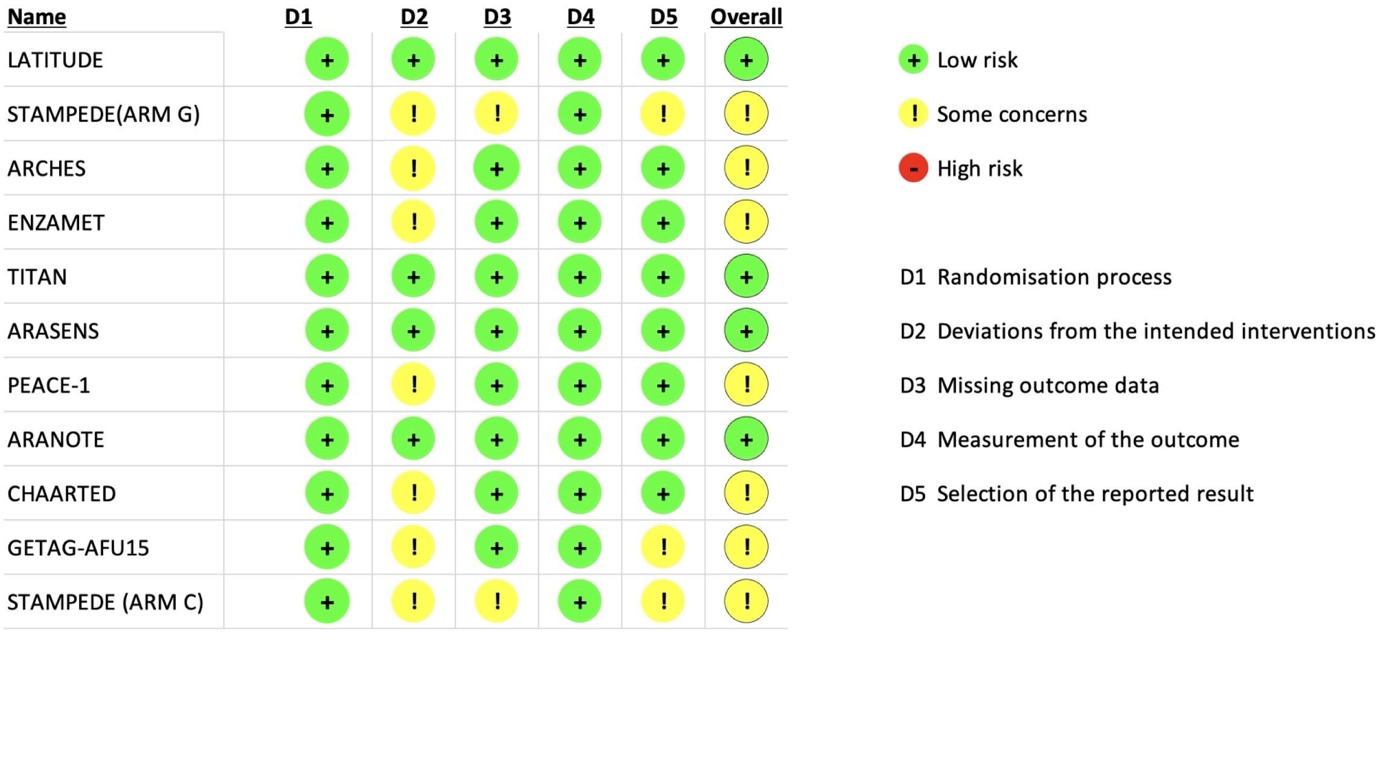
Figure S2**: Risk of Bias Assessment of the included trials.

Certainty of Evidence

This systematic approach categorizes evidence quality into four levels: very low, low, moderate, or high. Evidence quality was initially rated as high but could be downgraded based on critical evaluation of the following five domains: risk of bias, inconsistency, imprecision, indirectness, and publication bias. This process yielded a comprehensive assessment of the overall evidence certainty.

| **Outcomes** | **Certainty of the evidence (GRADE)** |
| --- | --- |
| Overall survival | **High** |
| Progression-free survival | **Moderate**^1^  due to indirectness |
| Adverse events (≥ grade 3) | **Moderate**^2,^  due to inconsistency |
| Specific Adverse events (hypertension, fatigue, bone fractures) | **Moderate**^2^ due to inconsistency |
| ^1^Different definitions of progression-free survival (clinical or radiographic)  ^2^Statistical heterogeneity based on meta-analysis results | |

**Supplementary Table 1:** Certainty of the evidence for each outcome based on the GRADE approach

**GRADE Working Group grades of evidence**

**High certainty:** We are very confident that the true effect lies close to that of the estimate of the effect.

**Moderate certainty:** We are moderately confident in the effect estimate; the true effect is likely to be close to the estimate of the effect, but there is a possibility that it is substantially different.

**Low certainty:** Our confidence in the effect estimate is limited; the true effect may be substantially different from the estimate of the effect.

**Very low certainty:** We have very little confidence in the effect estimate; the true effect is likely to be substantially different from the estimate of effect.


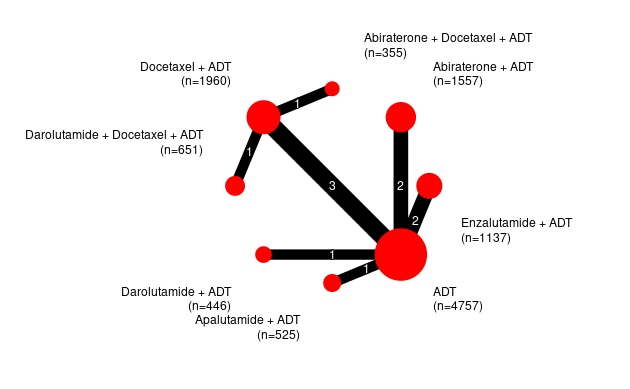


**Figure S3**. Network diagram. Lines demonstrate studies with direct comparisons and line thickness corresponds to number of connected studies.

Progression-free Survival

Subgroup analysis was performed for the age group below 65 to 70 years and equal to or above 70 to 75 years for PFS and OS for all doublet therapy interventions. In the age group below 65 to 70 years all interventions significantly improved PFS compared with SOC (Figure S4a). In the age group above 70 to 75 years enzalutamide plus ADT and abiraterone plus ADT significantly improved PFS compared with SOC (Figure S4b).

**
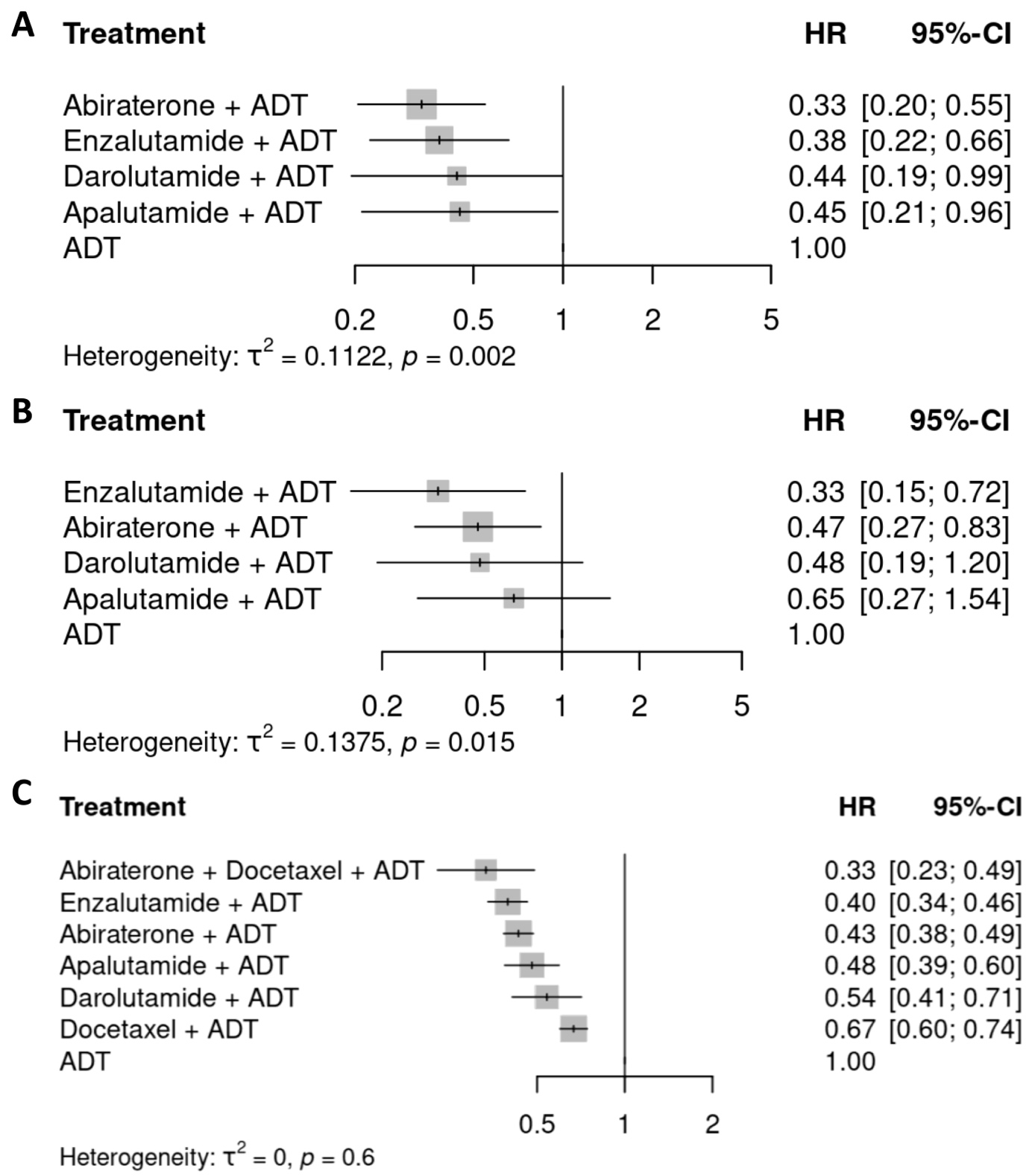
**

**Figure S4.** Progression-free Survival (PFS) Hazard Ratios (HRs) from Random Effects Network Meta-Analysis**. Figure S4a**. Random Effects Model for PFS in the age < 65-70 years subgroup within ARSI plus ADT interventions. **Figure S4b**. Random Effects Model for PFS in the age ≥ 70-75 years subgroup within ARSI plus ADT interventions. **Figure S4c**. Random Effects Model for PFS without the ARASENS trial.

Overall Survival

**
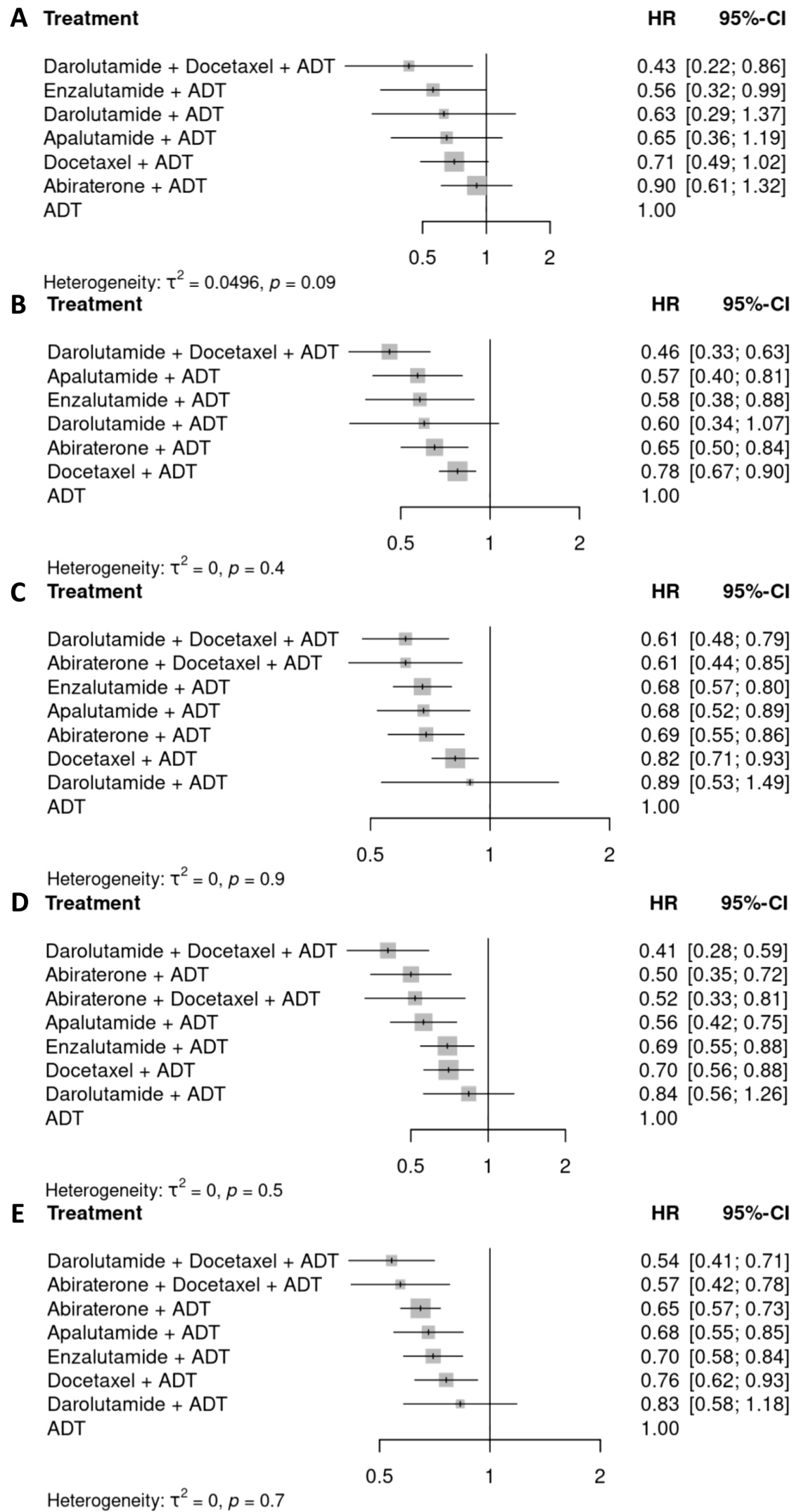
**

**Figure S5.** Overall Survival (OS) Hazard Ratios (HRs) from Random Effects Network Meta-Analysis. **Figure S5a**. Random Effects Model for OS in the age < 65-70 years subgroup. **Figure S5b.** Random Effects Model for OS in the age ≥ 70 -75 years subgroup. **Figure S5c**. Random Effects Model for OS in the ECOG 0 subgroup. **Figure S5d.** Random Effects Model for OS in the ECOG ≥ 1 subgroup. **Figure S5e.** Random Effects Model for OS in the *de novo* subgroup.


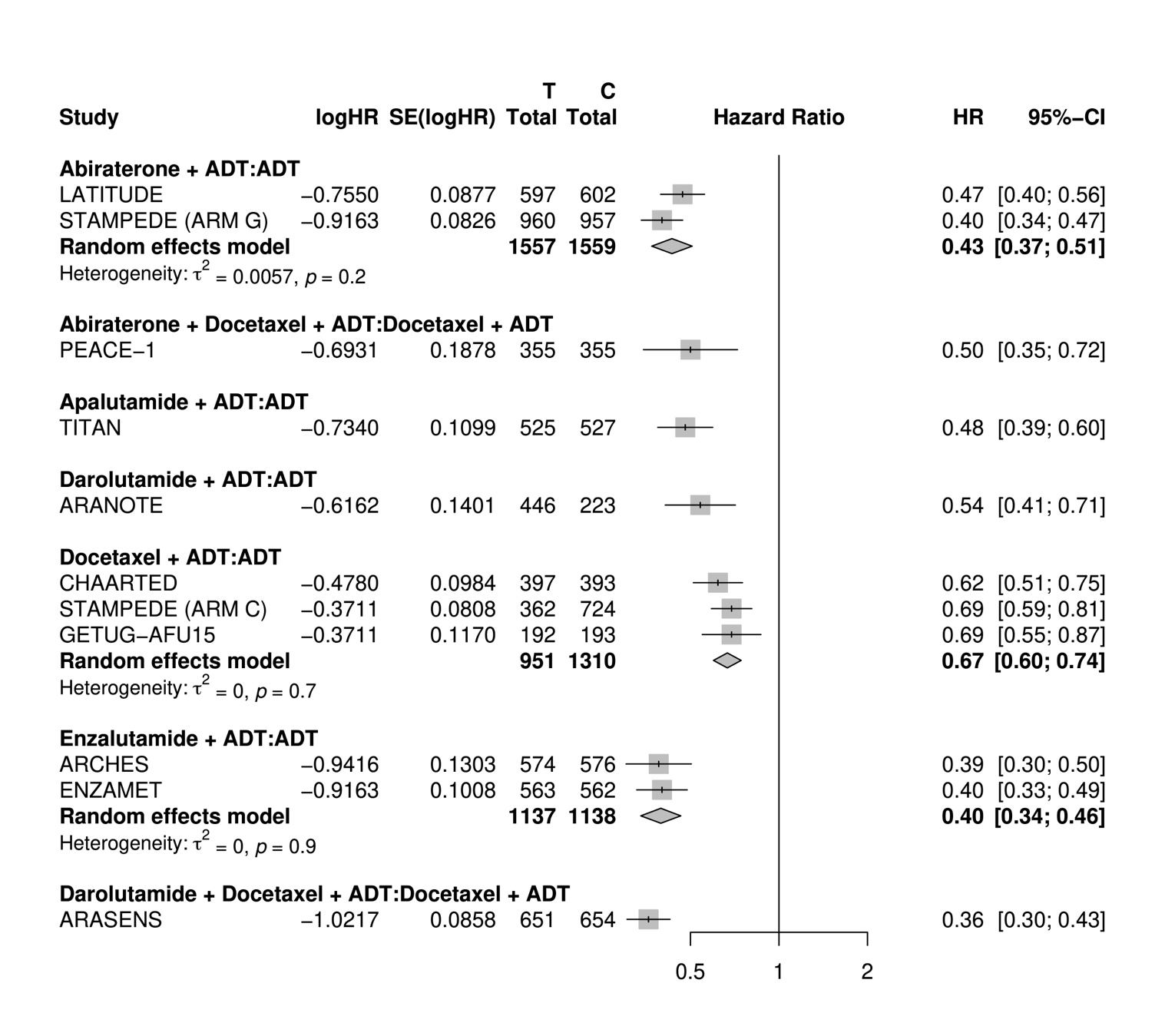


**Fig S6**. Progression-free Survival (PFS) Hazard Ratios (HRs) from Random Effects Network Meta-Analysis**.**


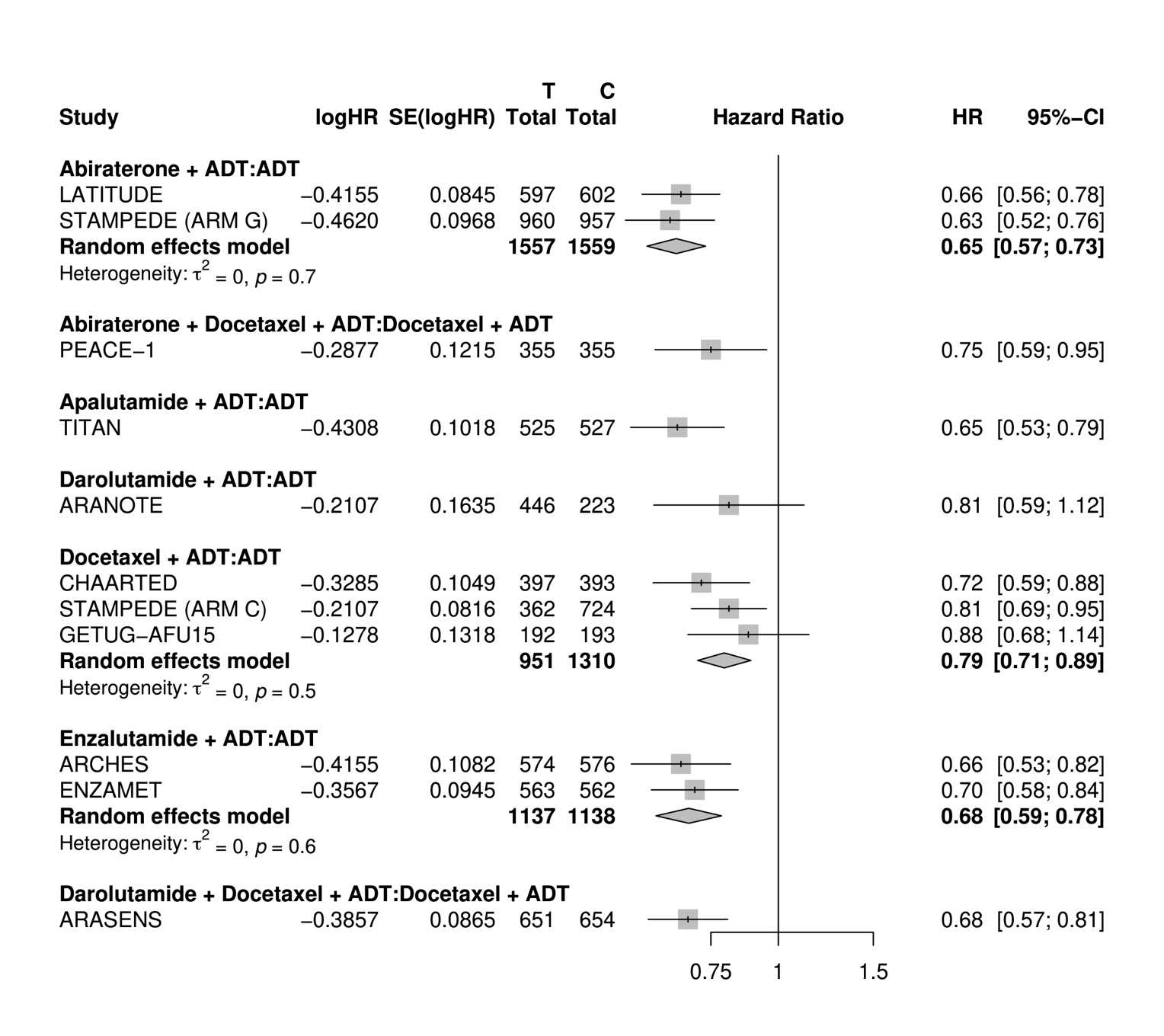
**Fig S7**. Overall Survival (OS) Hazard Ratios (HRs) from Random Effects Network Meta-Analysis
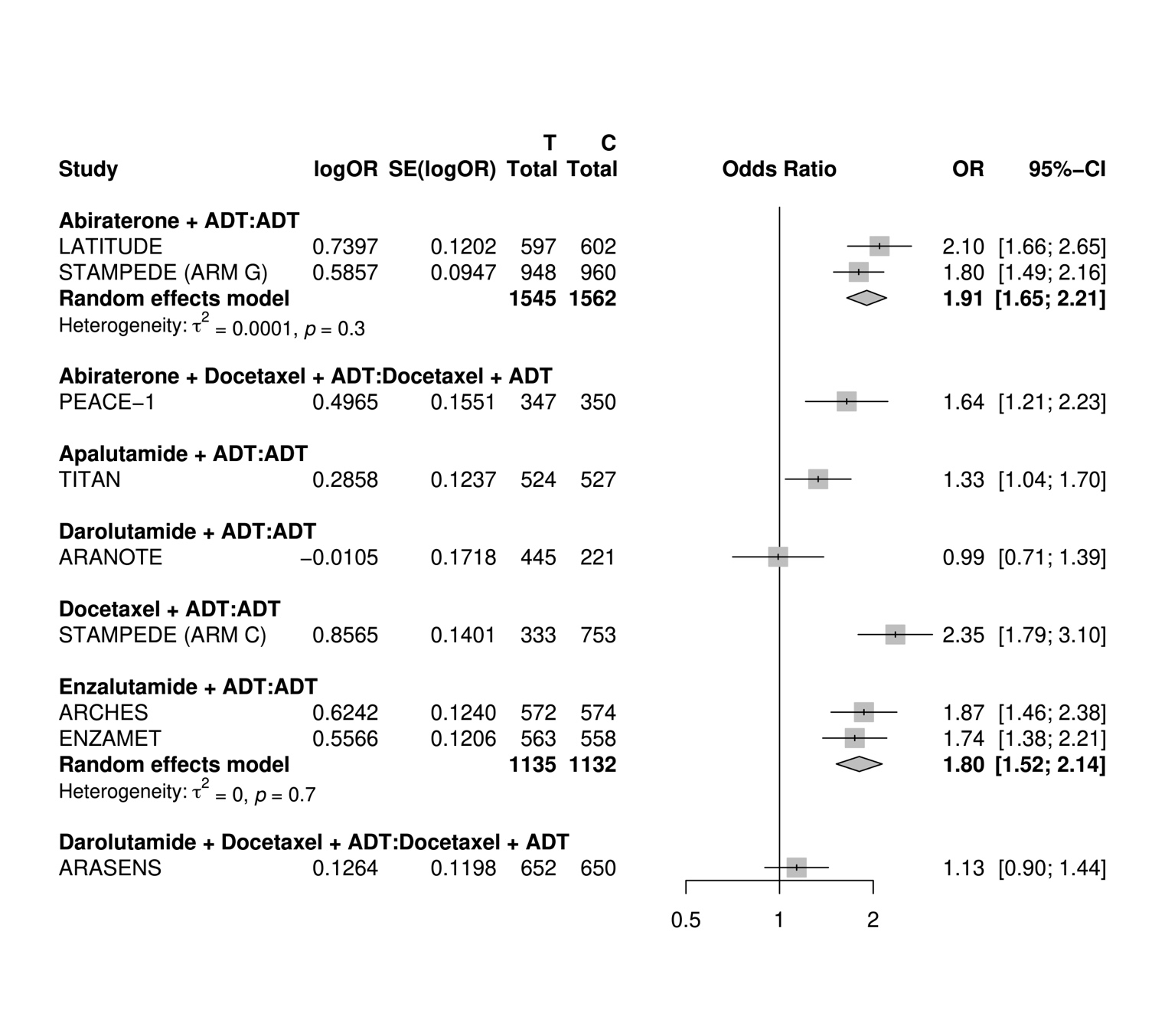
**Fig S8**. Treatment-Emergent Adverse Events (TEAEs) Odds Ratios (ORs) from Random Effects Network Meta-Analysis.
